# Comparing hydrodynamic performance in full-root and graft-inclusion Ross technique: An ex vivo study

**DOI:** 10.1016/j.xjon.2026.101917

**Published:** 2026-06-05

**Authors:** Nina Sophie Pommert, Thomas Puehler, Leonie Umminger, Buntaro Fujita, Igor Berezovets, Michael Scharfschwerdt, Mustafa Almanasef, Iliazbek Kazakbaev, Felix Hausbeck, Ioana Geisler, Barbara Linz, Stephanie Sellers, David Meier, Georg Lutter, Anas Aboud, Stephan Ensminger

**Affiliations:** a Department of Cardiac and Thoracic Vascular Surgery, University Medical Centre Schleswig-Holstein, Campus Luebeck, Luebeck, Germany; b DZHK (German Centre for Cardiovascular Research), Partner Site Hamburg/Kiel/Luebeck, Luebeck, Germany; c Cardiovascular Translational Lab, Centre for Heart Lung Innovation & Providence Research, University of British Columbia, Vancouver, British Columbia, Canada; d Department of Cardiology, Lausanne University Hospital and University of Lausanne, Lausanne, Switzerland; e Department of Cardiac Surgery, University Medical Centre Schleswig-Holstein, Campus Kiel, Kiel, Germany

**Keywords:** full-root replacement, graft inclusion, hemodynamics, in vitro, Ross, subcoronary, surgical technique

## Abstract

**Objectives:**

The Ross procedure is an attractive method for treating young patients with aortic valve disease, but the optimal surgical technique is still under discussion. This study aimed to evaluate the full-root and graft-inclusion Ross techniques in terms of their hydrodynamic performance ex vivo in a simulated circulation model.

**Methods:**

A total of 20 porcine autografts were created using either the full-root (n = 10) or graft-inclusion (n = 10) Ross technique. Hydrodynamic performance, including valvular opening morphology, transvalvular gradients, orifice area, and regurgitation volumes, were assessed under different resting and exercise conditions in an ex vivo mock-up circulation model.

**Results:**

Full-root porcine autografts exhibited significantly lower transvalvular pressure gradients and greater orifice areas compared with graft-inclusion autografts (mean pressure gradient: full root 3.6 vs graft inclusion 5.0 mm Hg, *P* = .007; effective orifice area full root 2.4 vs graft inclusion 2.0 cm^2^, *P* = .007). Valvular leakage was comparable between the 2 techniques, but graft inclusion showed a significantly lower valvular closing volume compared with the full-root Ross technique (full root: 7.9 vs graft inclusion: 4.8 mL, *P* = .002). The differences described were significant under all resting and exercise conditions examined.

**Conclusions:**

The full-root technique appears to be advantageous from a hydrodynamic perspective. It revealed lower gradients and greater orifice areas, and the surgical procedure is less complex to perform. However, larger closure volumes may be the reason for dilatation of the neo-aortic root. Therefore, further research on the prevention of neo-aortic root dilatation, focusing on the examination of the closing volume, is needed.

## Graphical Abstract

**Figure:**
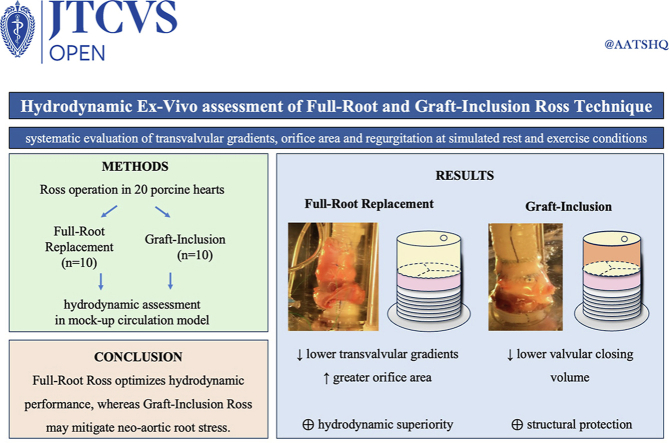

**Figure:**
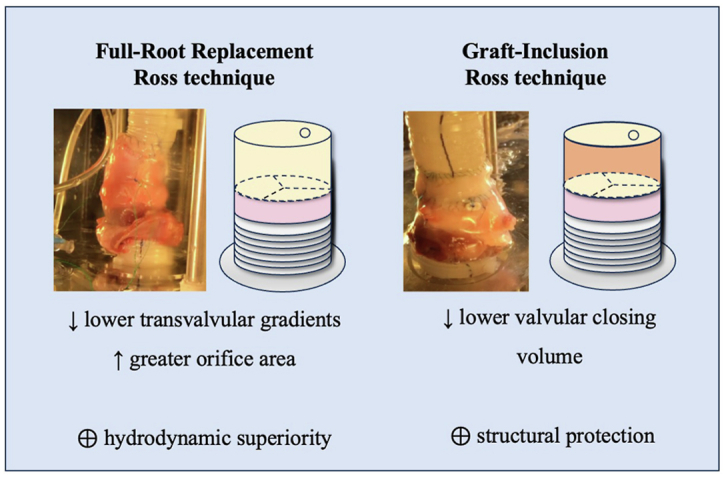
The FR Ross favors flow, and the GI technique ensures root protection.

**Central Message**

The FR Ross technique demonstrated superior hydrodynamic performance, whereas the GI technique showed lower closing volume, suggesting reduced forces on the neo-aortic root.

**Perspective**

The optimal surgical technique for the Ross procedure remains uncertain. Although the FR replacement technique demonstrated favorable hydrodynamic performance ex vivo, the GI technique showed lower closing volume that may be relevant for neo-aortic root durability.

The Ross procedure uses the autologous pulmonary valve as a “living” substitute for the diseased aortic valve, which makes it an attractive treatment option for young patients with aortic valve disease. The viable pulmonary substitute offers growth potential and regenerative capacity, allowing favorable long-term performance without the need for anticoagulation therapy.^1^, ^2^, ^3^ Since its publication by Ross^4^ in 1967, different modifications of the surgical technique have been undertaken. In the original subcoronary technique, the dissected pulmonary valve is implanted into the native aortic anulus. This technique ensures natural external protection of the pulmonary autograft by the native aortic root, maintaining nearly normal root mechanics.^5^^,^^6^ When performed by experienced surgeons, studies show excellent results with this challenging technique.^3^^,^^7^^,^^8^ The graft-inclusion (GI) technique is a slight modification of the original subcoronary technique, the difference being that it is not the dissociated pulmonary valve, but the entire right ventricular outflow tract cylinder that is continuously sutured into the aortic root without dissociating the graft at the coronary side. Compared with the subcoronary technique, the GI technique prevents distortion of the commissures, thus making it less technically demanding.^9^^,^^10^

The full-root (FR) technique is currently most widely used, because it reduces surgical complexity and preparation time. With this technique, the pulmonary autograft is used as a free-standing aortic root replacement with reinsertion of the coronary arteries.^11^ It is commonly paired with a replacement of the ascending aorta. Despite encouraging long-term results,^7^ this technique often leads to postoperative enlargement of the new aortic root, which in turn leads to autograft failure and the need for further surgery.^12^ To counteract this, different strategies to reinforce the pulmonary autograft have been introduced in the past, such as annular pericardial or Dacron strips, additional sutures or embedding the autograft in remnants of the native aortic wall, or Dacron prostheses.^7^^,^^8^

So far, the different surgical techniques have only been compared in clinical studies, mostly using retrospective study designs or register-based data sets. Exact analysis of their hydrodynamic properties has been lacking. Therefore, in this ex vivo study, we aimed to evaluate the 2 different Ross techniques, that is, FR replacement and GI technique, in terms of their hydrodynamic performance in an ex vivo setting.

## Materials and Methods

### Ethics Statement

This study was carried out in compliance with the German Animal Welfare Act and did not require approval by a local review board (TierSchG).

### Surgical Technique

**Figure 1:**
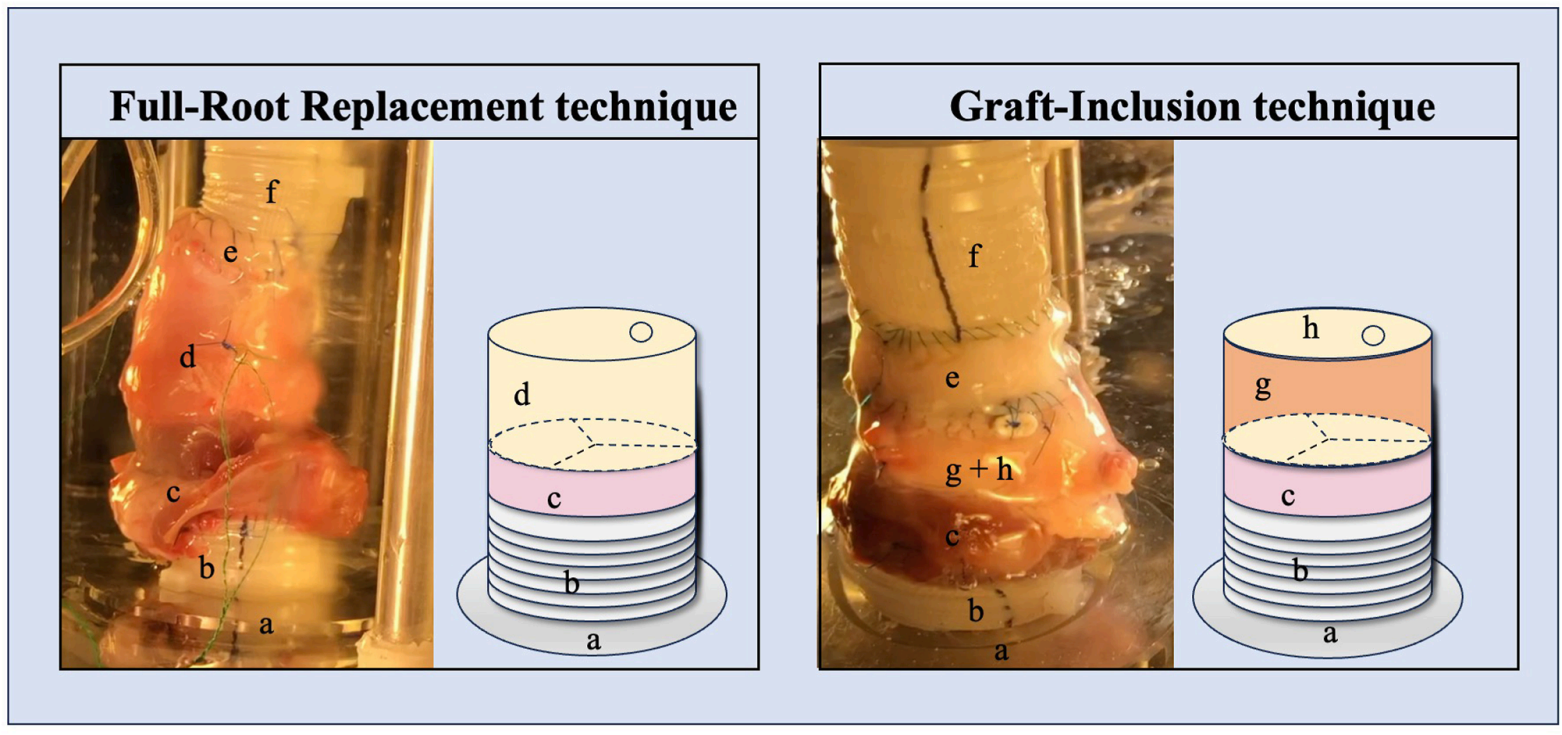
Ex vivo setup and schematic illustration of the FR and GI Ross techniques. A, Ventricular site of the pulse duplicator. B, Prosthetic connection to ventricular site. C, LVOT. D, Autograft (FR). E, Ascending aorta. F, Prosthetic connection to aortic site of the pulse duplicator. G, Aortic root. H, Autograft (pulmonary cylinder). For test purposes, the grafts were connected to Dacron prostheses on the aortic and ventricular sides.

We extracted aortic roots and pulmonary valves from porcine hearts and prepared native grafts using both the FR replacement and GI Ross techniques. A total of 20 native grafts were divided equally between the 2 techniques, with 10 subjects undergoing the FR technique and the remaining 10 subjects undergoing the GI technique. The procedures were conducted under the supervision of an experienced Ross surgeon. Details of the procedure have been published previously.^4^^,^^9^, ^10^, ^11^ A schematic illustration of the surgical technique and the ex vivo setup are shown in Figure 1. In both techniques, coronary artery reanastomosis was omitted in this ex vivo model for the purpose of hemodynamic evaluation.

As a first step, the aortic valve was excised, and the annulus was measured using Hegar dilators. The pulmonary autograft was then harvested at a subcommissural level with a remaining muscular rim of 3 mm below the pulmonary cusps. The pulmonary cusps were examined for fenestrations or damage. For the FR technique, the aortic root was excised and the full autograft was then implanted within the left ventricular outflow tract (LVOT) using 6 continuous 4/0 Prolene sutures, starting at the nadir of the leaflets and continuing circumferentially in a horizontal plane to the commissures. The distal anastomosis to the ascending aorta was a continuous Prolene 4/0 suture. No additional reinforcement strategies were applied within the FR technique.

For the GI technique, the proximal anastomosis was performed using single 4/0 Prolene sutures, paying special attention to the exact positioning of the commissures at 120°. After lowering the autograft into the LVOT, the distal anastomosis was performed at the level of the sinotubular junction using three 4/0 Prolene sutures to integrate the autograft into the aortic root. For implantation in the pulse duplicator, the grafts were connected to Dacron prostheses on the aortic and ventricular sides.

### Physiological Circulatory Simulation

Hydrodynamic performance was assessed under simulated circulation in a noncommercial pulse duplicator (Video 1). Technical details of the pulse duplicator have been described by Scharfschwerdt and colleagues.^13^ Physiological saline (0.9%) at room temperature was used as a perfusion solution. The measurements were conducted at a constant heart rate of 64 beats per minute. To simulate physiological rest and exercise levels, 4 different stroke volumes were tested. The mean pressure gradient (MPG), effective orifice area (EOA), closing volume (Vclose), and leakage volume of all grafts were evaluated under each condition.

**Figure 2:**
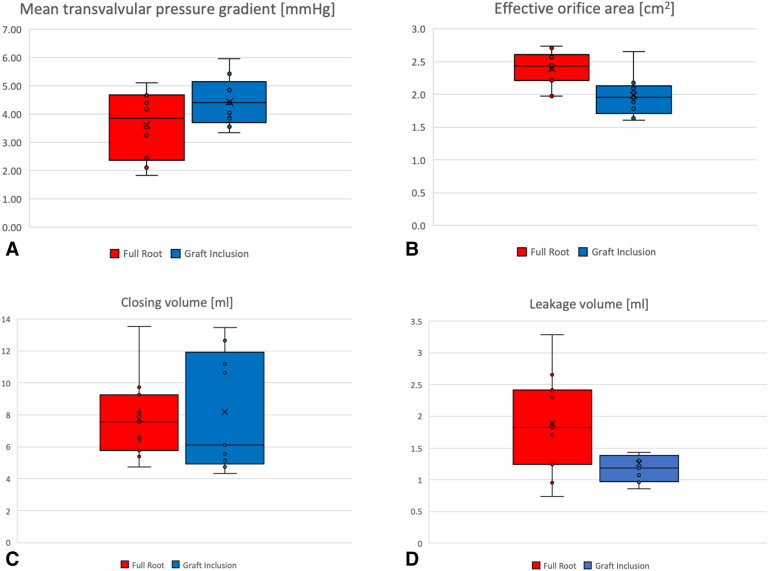
Valvular frames within the simulated cardiac cycle. Both techniques demonstrate symmetric leaflet conformation, with the commissures positioned at 120°, and a uniform opening movement. The maximum orifice area is nearly circular in shape.

The Vclose was defined as the amount of fluid that flows backward during the closure of the valve from the initiation of valve closure until the valve is fully closed (Vclose). The leakage volume refers to the backward flow after the valve has fully closed. Each measured value was recorded over 10 cardiac cycles. Video recordings of the valve during the test were taken with a high-speed camera over 1 cardiac cycle at 500 frames per second (Figure 2). All measurements were performed in accordance with the international norm for testing surgical prosthetic heart valves (ISO 5840: cardiovascular implants–cardiac valve prostheses).

### Statistical Analysis

Numeric variables were expressed as median ± interquartile range. A pairwise Wilcoxon rank-sum test was used to compare EOA, MPG, Vclose, and leakage volume between the 2 groups, with Bonferroni correction applied for multiple comparisons. To determine the study's sensitivity to detect differences between the surgical techniques, effect sizes were estimated using Cliff's delta. A sensitivity analysis was then performed to determine the minimum detectable effect size, given the sample size (n = 10 per surgical technique), α = 0.05, and power = 0.80. Wilcoxon rank-sum test with Bonferroni correction was conducted using SPSS Version 29.0.2.0 (20) (BM Corp). Cliff's delta and the sensitivity analysis were performed in R (Version 4.5.2 [2025-10-31]).

## Results

**Figure 3:**
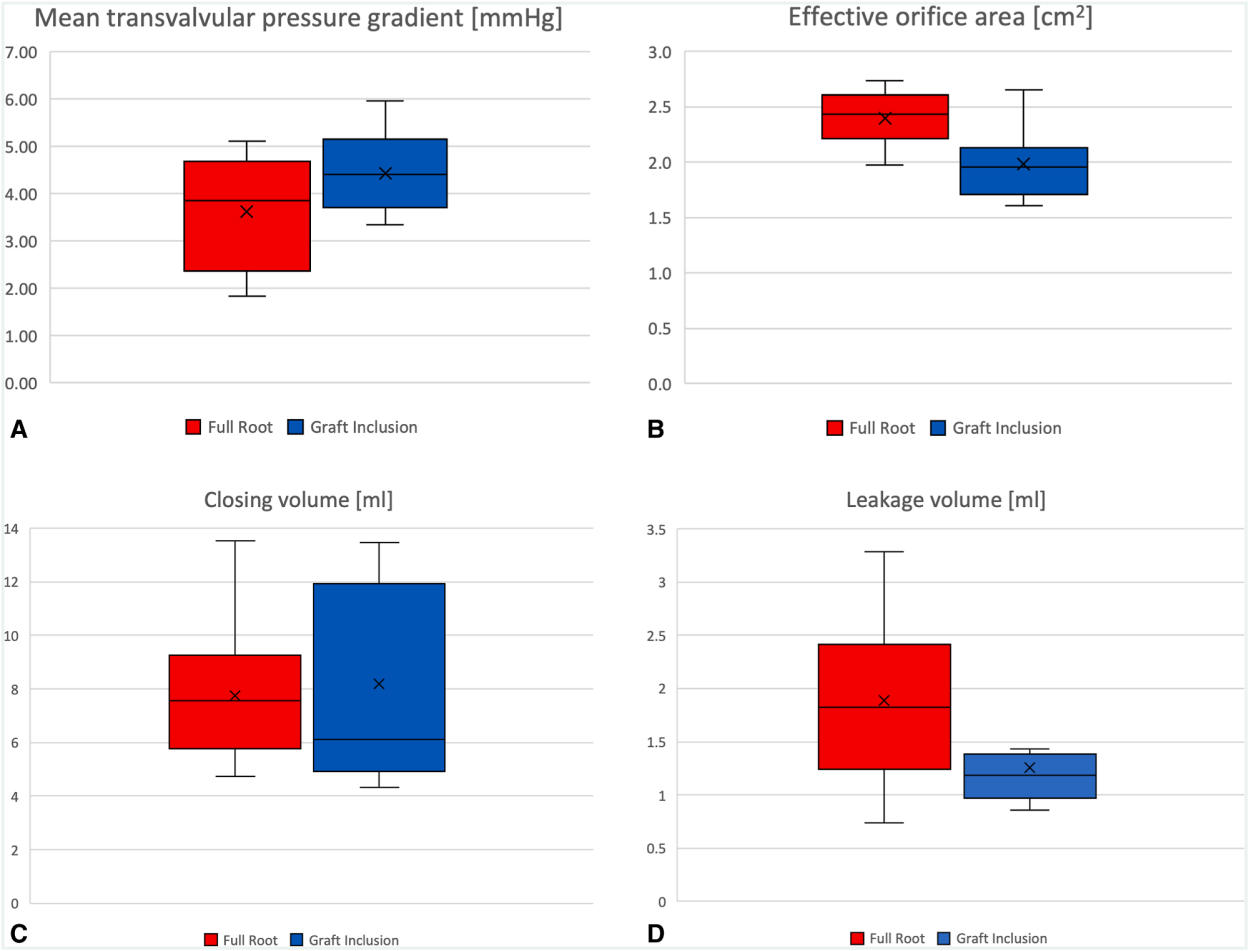
Ex vivo hydrodynamic performance of the FR and GI Ross techniques. A, Mean transvalvular pressure gradient in millimeters of mercury. B, Effective orifice area in centimeters squared. C, Vclose in milliliters. D, Leakage volume in milliliters. *Red*: FR technique. *Blue*: GI technique. Values given as mean value over 10 cardiac cycles in 10 porcine aortic roots per technique at moderate exercise load (stroke volume 70 mL, heart rate 64 bpm). Box plot legend: *horizontal line*: median; box: interquartile range; x: mean; whiskers: range (min-max).

A total of 20 native grafts were divided into the FR group (n = 10) and the GI group (n = 10). For an overall comparison, the different techniques were assessed at a moderate exercise level (stroke volume 70 mL, heart rate 64 bpm). The MPG, EOA, Vclose, and leakage volume are shown in Figure 3, *A-D* and Table 1.

**Table 1:** Hydrodynamic assessment of full-root and graft-inclusion Ross technique

Variable	FR	GI	*P* value
MPG [mm Hg]	3.6 (3.1-4.2)	5.0 (4.1-8.3)	.007
EOA [cm^2^]	2.4 (2.2-2.6)	2.0 (1.7-2.1)	.007
Vclose [mL]	7.9 (6.6-9.3)	4.8 (3.5-5.6)	.002
Leakage volume [mL]	3.0 (2.4-3.5)	1.8 (1.2-5.7)	.387

Values given as median and interquartile range over 10 cardiac cycles in 10 porcine aortic roots per technique at moderate exercise load (stroke volume 70 mL, heart rate 64 bpm). Wilcoxon rank-sum test. *FR*, Full-root; *GI*, graft-inclusion; *MPG*, mean pressure gradient; *EOA*, effective orifice area; *Vclose*, closing volume.

The mean transvalvular pressure gradient was significantly lower in the FR group compared with the GI group (FR: 3.6 [3.1-4.2] vs GI: 5.0 [4.1-8.3], *P* = .007; Figure 3, *A*). Accordingly, the effective orifice area was greater using the FR technique (FR: 2.4 [2.2-2.6], GI: 2.0 [1.7-2.1], *P* = .007; Figure 3, *B*). With regard to the Vclose from the beginning of diastole to valve closure, there was a significant difference in favor of the GI technique, in which the Vclose was significantly lower (FR: 7.9 [6.6-9.3], GI: 4.8 [3.5-5.6], *P* = .002; Figure 3, *C*). The leakage volumes after valve closure of the 2 surgical techniques were comparable (FR: 3.0 [2.4-3.5], GI: 1.8 [1.2-5.7], *P* = .387; Figure 3, *D*). However, a higher range of leakage volumes was observed in the GI technique (Figure 3, *D*).

**Figure 4:**
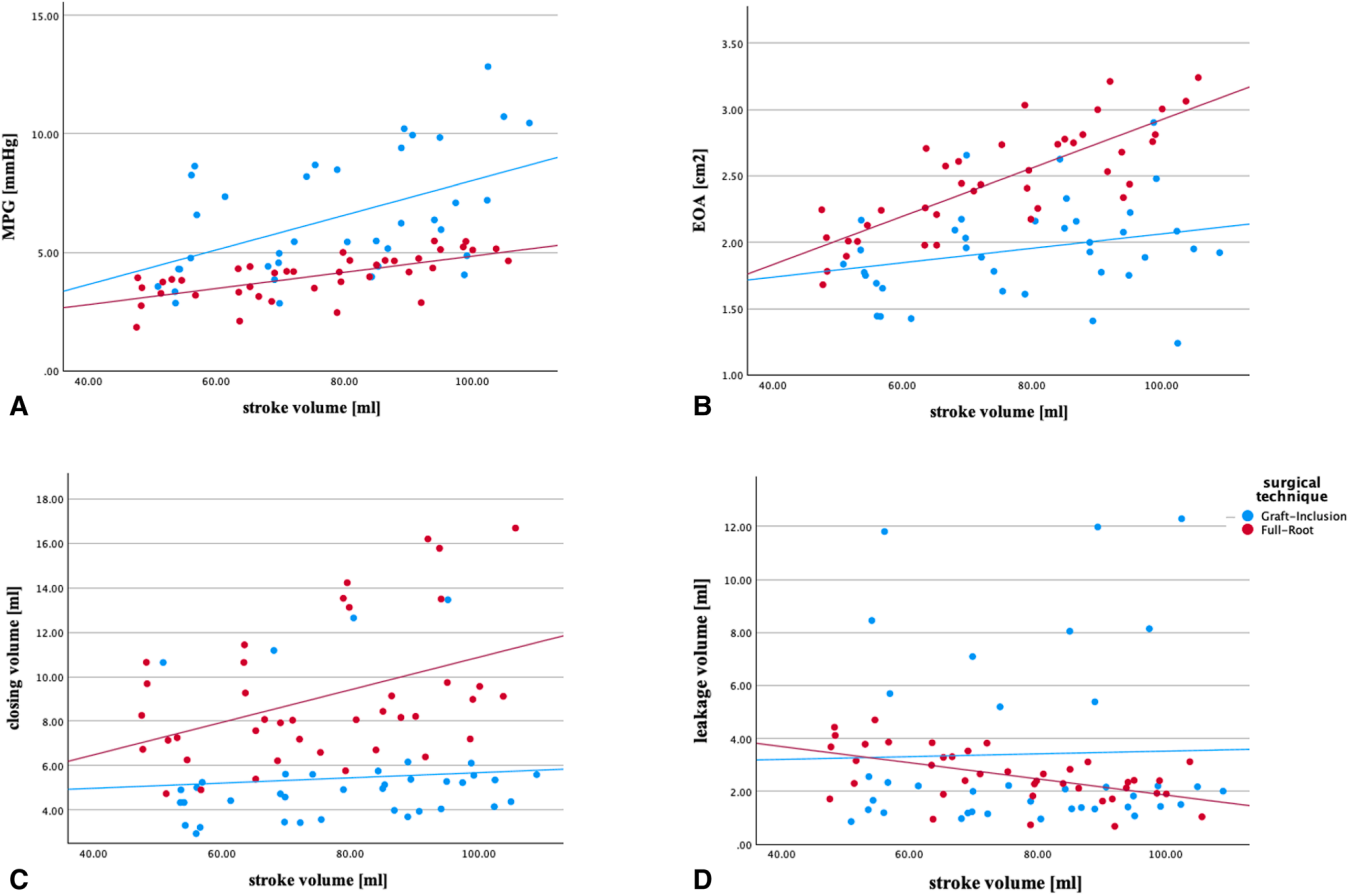
Ex vivo hydrodynamic assessment of FR and GI Ross techniques under simulated rest and stress. A, Mean transvalvular pressure gradient in millimeters of mercury. B, Effective orifice area in centimeters squared. C, Vclose in milliliters. D, Leakage volume in milliliters. *Red*: FR technique. *Blue*: GI technique. Scatter plots including group-specific linear regression lines.

The overall differences that were observed in the analysis at moderate exercise load were also seen when looking at each of the individual stroke volumes tested as shown in Figure 4 and Table 2. As expected, there was an increase in MPG, EOA, and Vclose with increasing stroke volume for both surgical techniques. Interestingly, the FR technique showed a significant decrease in leakage volume with increasing stroke volume (paired-samples test, *P* < .001) (Figure 4, *D*).

**Table 2:** Hydrodynamic assessment of full-root and graft-inclusion Ross techniques under simulated rest and stress

Variable	FR	GI	*P* value
MPG [mm Hg]			
Condition 1	3.5 (3.0-3.8)	4.5 (3.5-7.6)	.017
Condition 2	3.6 (3.1-4.2)	5.0 (4.1-8.3)	.007
Condition 3	4.3 (3.9-4.7)	5.9 (5.0-9.9)	.003
Condition 4	5.1 (4.6-5.3)	7.1 (5.4-10.6)	.013
EOA [cm^2^]			
Condition 1	2.0 (1.8-2.2)	1.7 (1.4-1.9)	.013
Condition 2	2.4 (2.2-2.6)	2.0 (1.7-2.1)	.007
Condition 3	2.7 (2.4-2.9)	2.1 (1.8-2.2)	<.001
Condition 4	2.8 (2.5-3.1)	2.1 (1.9-2.4)	.001
Vclose [mL]			
Condition 1	7.2 (6.5-9.0)	4.4 (3.3-5.1)	.008
Condition 2	7.9 (6.6-9.3)	4.8 (3.5-5.6)	.002
Condition 3	8.3 (7.7-13.2)	5.2 (4.0-5.9)	<.001
Condition 4	9.6 (8.5-15.9)	5.3 (4.3-5.8)	<.001

Values given as median and interquartile range over 10 cardiac cycles in 10 porcine aortic roots per technique at low to high exercise load (condition 1: stroke volume = 53 mL, condition 2: stroke volume = 70 mL, condition 3: stroke volume = 85 mL, condition 4: stroke volume = 99 mL). Wilcoxon rank-sum test. *FR*, Full-root; *GI*, graft-inclusion; *MPG*, mean pressure gradient; *EOA*, effective orifice area; *Vclose*, closing volume.

Confirming significant differences between the techniques for mean transvalvular gradient, effective orifice area, and Vclose, Cliff's delta indicated large effect sizes for these parameters (δ MPG = 0.70, δ EOA = −0.70 and δ Vclose = −0.78). In contrast, no robust difference was found for the leakage volume (δ = −0.24), with a CI including zero. The sensitivity analysis indicated that given the small sample size, the study was only powered to detect medium-to-large effects (Cliff's δ ≈ 0.55-0.70). Consequently, smaller effects may not have been detectable.

## Discussion

The long-term clinical success of the Ross procedure makes it a compelling option for the treatment of aortic valve disease in young patients.^1^, ^2^, ^3^^,^^7^ However, subsequent aortic root and autograft pathologies have emerged as major limitations of the procedure and have led to ongoing discussions regarding its indication and techniques.^12^^,^^14^ As of today, the majority of scientific work on the Ross procedure almost exclusively consists of clinical trials. Hydrodynamic tests under standardized conditions, as required for valve prostheses, are lacking. This ex vivo study therefore aimed to investigate the hydrodynamic performance of the Ross procedure in simulated circulation. The primary objective was to conduct a comparative analysis of 2 prevalent Ross techniques: FR and GI.

**Figure 5:**
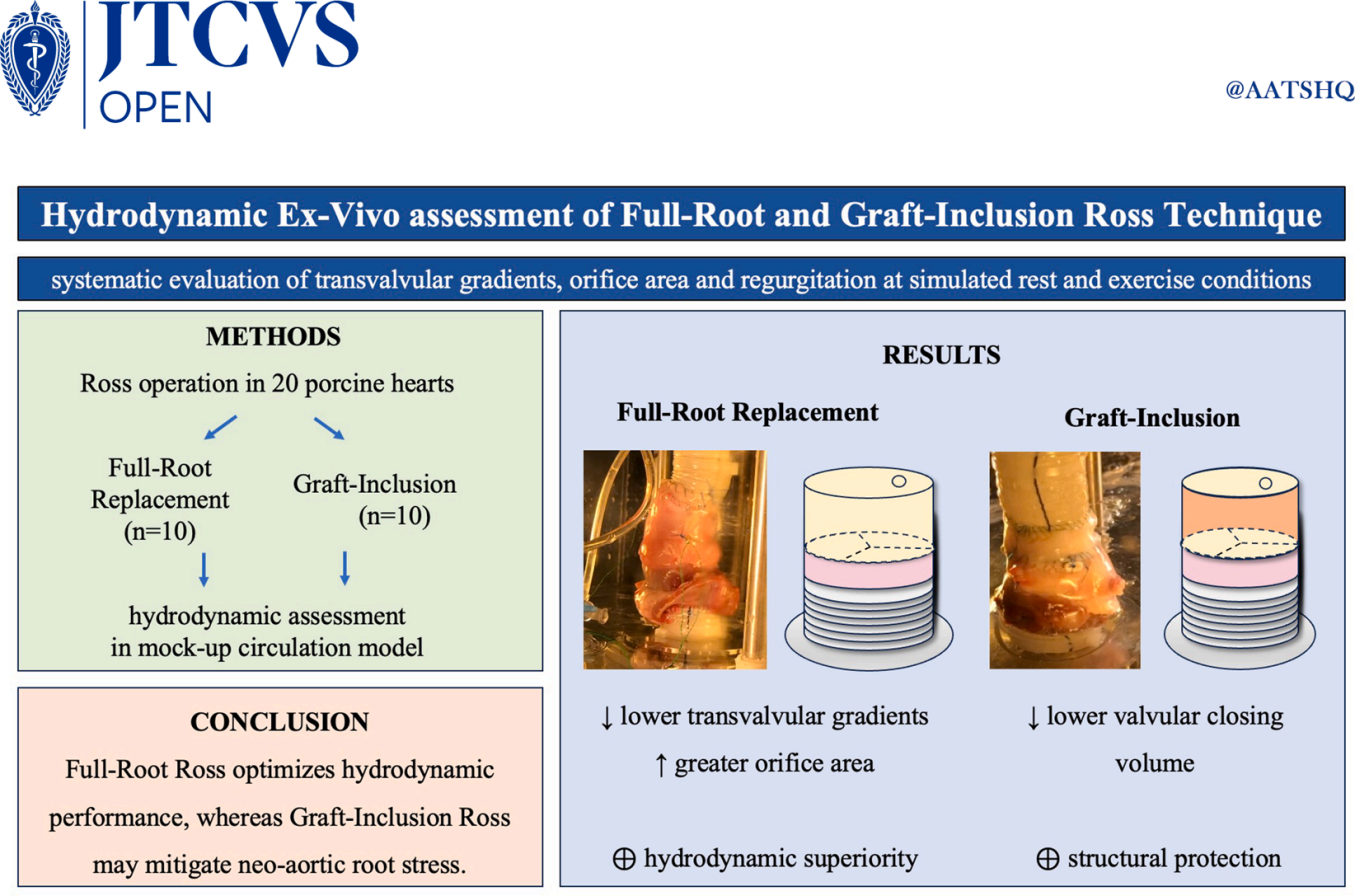
Graphical Abstract. Summary of the study design, principal results, and main conclusion.

The main findings of our ex vivo hydrodynamic evaluation of the FR and GI Ross techniques are as follows (Figure 5):

1. Porcine autografts operated using the FR Ross technique exhibit a significantly lower mean transvalvular pressure gradient and a greater effective orifice area compared with those operated using the GI Ross technique.
2. No significant difference in valvular leakage was observed between the 2 surgical techniques, despite a wider range of values in the GI technique.
3. The GI Ross technique leads to a significantly lower valvular Vclose than the FR Ross technique.
4. The differences described are significant both for the analysis of the mean values and for all resting and physiological stress conditions examined.

### Transvalvular Gradients, Orifice Area, and Valvular Leak

Across all stress conditions, the EOA for the FR technique was approximately 0.5 cm^2^ higher than for the GI technique. Correspondingly, the transvalvular MPG was approximately 2 mm Hg lower. In a preliminary study in our laboratory, porcine aortic valves of comparable size were evaluated under identical test conditions. This showed an EOA of approximately 1.8 cm^2^ and transvalvular MPG of 6.8 mm Hg at low to moderate exercise load in native porcine aortic valves, which supports favorable flow characteristics for both Ross techniques.^15^

Clinical studies confirm the advantageous hemodynamics of the Ross procedure. In a comparison with homografts and xenografts in aortic position using magnetic resonance velocity mapping, pulmonary autografts were found to have the largest EOA and the best overall hemodynamic performance.^16^ An echocardiographic examination of 381 Ross patients also confirmed the significant difference in EOA and transvalvular gradients in favor of the FR technique.^17^ Moreover, the authors described clinically relevant regurgitation to be rare and independent of the surgical technique, supporting our ex vivo results.^17^ The wider range of the leakage volume in the GI technique group could be explained by the surgical complexity of the procedure, which makes it more difficult to reproduce the exact same anatomy and prone to variability.

### Valvular Morphology

The autograft function depends on the anatomy, integrity, and intact 3-dimensional geometric structure of the aortic root.^12^ In this study, under optimal conditions within the laboratory, it was possible to pay special attention to surgical precision in detail, such as the exact placement of the commissures at 120° and the correct implantation plane. For both surgical techniques, this resulted in a uniform valve opening movement with an approximately triangular maximum orifice area. Under real surgical conditions in the operating room with sometimes limited view, time pressure, and anatomic abnormalities within the diseased aortic root, this precise ex vivo surgical technique may have to be modified, making it less reproducible, especially for the GI technique. The training and experience of the surgeon are therefore of the utmost importance.^12^^,^^18^

### Neo-Aortic Root Dilatation and Closing Volume

The adaptation of the pulmonary autograft to the volume and pressure conditions in the LVOT and aortic root is crucial for a durable success of the Ross procedure. Using the GI technique, as well as the original subcoronary technique, the pulmonary autograft is sutured into the native aortic root for natural external reinforcement, maintaining normal root dynamics and preventing long-term dilatation. However, this technique is surgically demanding, less reproducible, and restricted to a few experienced surgeons.^12^^,^^18^ With the surgically simpler FR technique, there is clinical concern about dilatation of the neo-aortic root over time. Studies confirm neo-aortic root dilatation and subsequent valvular insufficiency in cases of unsupported FR technique, causing morbidity and requiring further surgery.^14^^,^^18^ This observation is likely due to fundamental differences in tissue architecture between the pulmonary artery and the aortic wall, with the pulmonary wall being thinner and more distensible. From a hydrodynamic perspective, an increased valvular Vclose and the associated increased retrograde diastolic flow may contribute to progressive neo-aortic dilation by transiently elevating cyclic wall stress in the aortic root.

In this study, the Vclose measured for the FR technique (9 mL) exceeded that of the native porcine aortic valve reported in a previous study undertaken at our laboratory (<3 mL) by more than 3-fold.^19^ Therefore, given the forces involved, one would expect rapid dilatation of the aortic root in our experimental FR grafts. In contrast to current clinical practice, no special reinforcement strategies, such as annular banding or autograft wrapping, including the emerging Ross personalized external aortic root support (PEARS) technique, were applied in this assessment. In further studies, it would be of interest to analyze whether these strategies have an impact on the Vclose. However, in this study, the LVOT was connected to a Dacron prosthesis a few millimeters below the proximal autograft suture line. Therefore, we assume that reinforcement with an annular band alone would have little impact on changes in the Vclose; however, it should be emphasized that current literature does not provide evidence for a causal relationship between an increased Vclose and neo-aortic dilation, and this proposed mechanism should be regarded as a theoretical consideration.

### Clinical Perspective

Aortic root dilatation after unsupported FR technique surgery is well known. The unsupported technique is therefore rarely used now in a clinical context.^12^^,^^18^^,^^20^ An emerging new technique is the combination of a PEARS procedure with the Ross operation (Ross-PEARS procedure), which seems to be a suitable technique to stabilize the aortic root when using the FR technique.^21^ Furthermore, several studies have demonstrated no significant differences in overall survival or autograft reoperation rates between reinforced FR replacement and the subcoronary Ross technique in children or adults.^7^^,^^14^ The GI technique used in this study is rarely used overall, but is comparable to the subcoronary technique, because in both techniques the autograft is supported by the native aortic root.

### Limitations

For this study, operations were performed on porcine hearts under optimal conditions in the laboratory. Findings from a porcine model cannot be directly transferred to the human aortic root. With regard to biomechanical properties, direct comparative data between porcine and human roots in younger human cohorts eligible for the Ross procedure are limited. Available evidence suggests that young porcine aortic tissue shows good correspondence with human tissue aged less than 60 years,^22^ but direct comparative studies of the aortic root remain scarce. This should be considered when interpreting the translational relevance of the present findings.

In addition, circulation was simulated, even though it complied with the international norm for testing surgical prosthetic heart valves. The simulation of exercise and rest levels in this simulated circulation has been obtained by adjusting the stroke volume, whereas in the human body, an increase in heart rate would also be expected. It is hypothesized that an increase in heart rate might reduce the regurgitation as the diastolic time decreases. However, higher heart rates may be associated with changes in transvalvular pressure gradients and flow acceleration. These interacting factors were not captured in the present experimental setup and warrant further investigation. In contrast to daily clinical practice, no reinforcement strategies were used that could have influenced the parameters measured. Furthermore, this study analyzed the hydrodynamic properties directly after surgery, and long-term performance has not been investigated. The sample size of 10 grafts per surgical technique, which was determined considering the effort required for the surgical preparation, limits statistical power, because only large effects were detectable according to the sensitivity analysis. Therefore, the study should be considered an exploratory investigation.

Although transvalvular gradients, orifice area, and Vclose reached statistical significance in this study, the absolute differences between groups were small, and their clinical relevance remains uncertain. The findings should be interpreted cautiously, because this study provides primarily mechanistic insights under controlled conditions rather than evidence to guide surgical decision-making.

## Conclusions

Compared with the GI technique, the FR replacement Ross technique appears to be advantageous from a hydrodynamic perspective due to its lower gradients and greater orifice area, as well as its simpler surgical feasibility. However, the larger closure volume may be an important factor triggering dilatation of the neo-aortic root, which is frequently observed after surgery using the FR technique. Therefore, further research into the prevention of neo-aortic root dilatation should also focus on examination of the Vclose.

## Conflict of Interest Statement

The authors reported no conflicts of interest.

The *Journal* policy requires editors and reviewers to disclose conflicts of interest and to decline handling or reviewing manuscripts for which they may have a conflict of interest. The editors and reviewers of this article have no conflicts of interest.

## Supplementary Data

Video 1 Experimental setup of a full-root Ross autograft in the pulse duplicator. Video available at: https://www.jtcvs.org/article/S2666-2736(26)00340-2/fulltext.

## References

[1] Sievers H.H., Stierle U., Charitos E.I. (2016). A multicentre evaluation of the autograft procedure for young patients undergoing aortic valve replacement: update on the German Ross Registry†. Eur J Cardiothorac Surg.

[2] Mastrobuoni S., de Kerchove L., Solari S. (2016). The Ross procedure in young adults: over 20 years of experience in our institution. Eur J Cardiothorac Surg.

[3] David T.E., Ouzounian M., David C.M., Lafreniere-Roula M., Manlhiot C. (2019). Late results of the Ross procedure. J Thorac Cardiovasc Surg.

[4] Ross D.N. (1967). Replacement of aortic and mitral valves with a pulmonary autograft. Lancet.

[5] Lancellotti P., Tribouilloy C., Hagendorff A. (2010). European Association of Echocardiography recommendations for the assessment of valvular regurgitation. Part 1: aortic and pulmonary regurgitation (native valve disease). Eur J Echocardiogr.

[6] Luciani G.B., Lucchese G., De Rita F., Puppini G., Faggian G., Mazzucco A. (2012). Reparative surgery of the pulmonary autograft: experience with Ross reoperations. Eur J Cardiothorac Surg.

[7] Aboud A., Charitos E.I., Fujita B. (2021). Long-term outcomes of patients undergoing the ross procedure. J Am Coll Cardiol.

[8] Fujita B., Aboud A., Sievers H.H., Ensminger S. (2021). State-of-the-art: insights from the Ross registry. JTCVS Tech.

[9] McGiffin D.C., Kirklin J.K. (1997). Homograft aortic valve replacement: the subcoronary and cylindrical techniques. Oper Tech Thorac Cardiovasc Surg.

[10] Ozturk M., Tongut A., Hanabergh S.S., Yerebakan C., El Khoury G., d'Udekem Y. (2022). The Ross procedure with the inclusion technique. Oper Tech Thorac Cardiovasc Surg.

[11] Forcillo J., Cikirikcioglu M., Poirier N., El-Hamamsy I. (2014). The Ross procedure: total root technique. Multimed Man Cardiothorac Surg.

[12] Sievers H.H., Hanke T., Stierle U. (2006). A critical reappraisal of the Ross operation: renaissance of the subcoronary implantation technique?. Circulation.

[13] Scharfschwerdt M., Misfeld M., Sievers H.H. (2004). The influence of a nonlinear resistance element upon in vitro aortic pressure tracings and aortic valve motions. ASAIO J.

[14] Weixler V., Murin P., Samman B. (2025). Subcoronary versus full-root Ross procedure for the paediatric population: an early-to-midterm bicentric experience. Eur J Cardiothorac Surg.

[15] Saisho H., Scharfschwerdt M., Schaller T. (2021). An ex vivo evaluation of two different suture techniques for the Ozaki aortic neocuspidization procedure. Interact Cardiovasc Thorac Surg.

[16] Torii R., El-Hamamsy I., Donya M. (2012). Integrated morphologic and functional assessment of the aortic root after different tissue valve root replacement procedures. J Thorac Cardiovasc Surg.

[17] Boehm J.O., Botha C.A., Hemmer W. (2004). Hemodynamic performance following the Ross operation: comparison of two different techniques. J Heart Valve Dis.

[18] David T.E., Omran A., Ivanov J. (2000). Dilation of the pulmonary autograft after the Ross procedure. J Thorac Cardiovasc Surg.

[19] Erasmi A., Sievers H.H., Scharfschwerdt M., Eckel T., Misfeld M. (2005). In vitro hydrodynamics, cusp-bending deformation, and root distensibility for different types of aortic valve-sparing operations: remodeling, sinus prosthesis, and reimplantation. J Thorac Cardiovasc Surg.

[20] El-Hamamsy I., Vricella L.A. (2024). Late pulmonary autograft dilation: can we make a good operation great? The tailored approach. Semin Thorac Cardiovasc Surg Pediatr Card Surg Annu.

[21] Hebala M., Nassar M., Austin C., Kenny L. (2025). Personalized external root support for the pulmonary autograft in the aortic position: the ROSS-PEARS procedure. Oper Tech Thorac Cardiovasc Surg.

[22] de Beaufort H.W.L., Ferrara A., Conti M. (2018). Comparative analysis of porcine and human thoracic aortic stiffness. Eur J Vasc Endovasc Surg.

